# Supplementary material for: Role of intrapulmonary lymph nodes in patients with NSCLC and visceral pleural invasion. The VPI 1314 multicenter registry study protocol
Source: PLoS One. 2023 May 4;18(5):e0285184. doi: 10.1371/journal.pone.0285184 (PMC10159114; doi:10.1371/journal.pone.0285184)
Supplement: S1 File — (PDF) [file pone.0285184.s002.pdf]

Research plan/Protocol for HRO:
Further use of biological material and health-related personal data for research pursuant to Articles 32 and 33 HRA

Title of the research project

Role of intrapulmonary lymph nodes in patients with NSCLC and visceral pleural invasion. The VPI 1314 multicenter registry study protocol

Name and address of the project leader
Fabrizio Minervini, MD, PhD
Staff surgeon
Department of Thoracic Surgery
Cantonal Hospital Lucerne
Spitalstrasse
6000 Lucerne 16
+41412056068
fabriziominervini@hotmail.com

If applicable: Name and address of the sponsor
Project leader

Confirmation of the project leader and the sponsor
With my signature, I attest that all information in this protocol is correct and that I will comply with the information I have given and with national legislation, namely data protection law.

Project leader:

Lucerne, 14.12.2022
Place, date ______________________ Signature

If applicable and not identical with the project leader: Sponsor:

______________________ ______________________
Place, date Signature

Abbreviations

NSCLC: non small cell lung cancer

OS: overall survival

DFS: disease free survival

CT: computed tomography

PET: positron emission tomography

1. Background

Lung cancer is the leading cause of cancer related death worldwide. More than 80% of all lung tumors are Non-Small Cell Lung Cancers (NSCLC). Lymph node staging has a prognostic value and is crucial to establish the optimal treatment strategy in individual patients. Computed tomography (CT) of the chest is the most commonly used imaging technique to assess the primary tumor along with intra thoracic metastases. However, CT imaging has several limitations when used as the single imaging modality (sensitivity and specificity of 55% and 81%, respectively).PET imaging, especially when combined with CT, plays an eminent role in the evaluation of intrathoracic and extra thoracic metastases and is therefore recommended preoperatively for most patients suspected of having lung cancer (sensitivity and specificity of 77% and 86% respectively, when evaluating for mediastinal metastasis).Confirming mediastinal involvement detected in PET is a key point in the treatment of lung cancer.
It remains unknown whether dissecting the intrapulmonary lymph nodes (stations 13 and 14) is necessary for accurate staging and prognostication. Although suggested by several guidelines, these peripheral lymph nodes are not routinely examined in clinical routine for several reasons. With the emergence of new and effective adjuvant treatment options, including immunotherapy and targeted agents, peripheral lymph node sampling could potentially be important. Moreover, the prognostic significance of the visceral pleural invasion is controversial. Some studies showed a negative impact on OS and DFS in patients with histologic proved visceral pleura invasion (VPI is defined as invasion beyond the elastic layer —>PL1, including invasion to the visceral pleural surface—>PL2).
The mechanism to explain this negative effect is not fully understood.
Given that the visceral pleura is very rich in lymphatic vessels, with an intercommunicating "network" arranged over the lung surface and penetrating into the lung parenchyma to join the bronchial lymph vessels with drainage to the various hilar nodes, we assume that the worse OS and DFS observed in these patients could be explained with the presence of metastatic lymph nodes (Station 13-14) that are not routinely examined.

2. Objectives

The aim of this multicenter prospective study is to evaluate the incidence of intrapulmonary lymph nodes metastases and the possible relation with visceral pleural invasion. Identifying patients with lymph node station 13 and 14 metastases and/or a link between visceral pleural invasion and presence of micro/macro metastases in intrapulmonary lymphnodes may have an impact on decision-making process.

3. Design

This is a retrospective multicenter study based on ad-hoc created prospectively database. Data will be collected through chart reviews from patient electronic medical record and institutional databases to identify patients who underwent elective lobectomy/bilobectomy/segmentectomy for NSCLC and lymphadenectomy.

4. Origin of the data/biological material

The patients to be included in the study will be identified through a prospectively entered database (start 1.1.2023 until the reach of the minimum sample size)
The following variables will be included in the data collection form:
Age Sex (M/F/Other)
Cardiac comorbidity (Y/N)
Pulmonary comorbidity (Y/N)
Pre-op FEV1 (L,%)
Pre-op DLCO (%)
Pre-op PET (Y/N)

Pre-op mediastinal Staging (Y/N) if Y (EBUS/EUS, Mediastinoscopy, others + biopsied stations level)
Surgical access (Open, VATS, Robotic)
Site (RUL, MLL, RLL, LUL, LLL)
Type of resection (Segmentectomy, Lobectomy, Bilobectomy)
Length of stay
Tumor Size
Histology
Extent of lymphadenectomy
cTNM
pTNM
PL status
Metastatic Lymphnodes N1 (10, 11, 12, 13, 14)
PDL1 (%)
EGFR Mutation (Y/N/U)
ALK Mutation (Y/N/U)
Post-op complication (Y/N)
Re-op (Y/N) if Y Reason
Adjuvant treatment (Y/N) (Chemotherapy, Radiation, Chemo-Radiation, Immunotherapy, Chemo
Immunotherapy, Chemo-Radio-Immunotherapy, Radio-Immunotherapy)
- If Chemotherapy: type of Chemo
- If Radiotherapy: regimen
Recurrence (Y/N) if Y: months after surgery
Death (Y/N) if Y: months after surgery

All the data will be collected from patients charts, pathology reports, surgery reports and outpatients clinic reports (routine appointments)

5. Inclusion criteria

Anatomical resection for NSCLC <3 cm (lobectomy, bilobectomy, segmentectomy)
R0 resection
General consent signed

6. Exclusion criteria

Prior or synchronous lung cancer
pN2
Pneumonectomy
R1/R2 resection
M1
Neoadjuvant treatment

7. Information and consent of participants
All patients have signed the general consent of the hospital (copy enclosed).
<u>If general consent is not available or not signed, we will consider it as exclusion criteria.</u>

8. Scientific methods and sample size

<u>Primary Outcomes</u>

- Overall incidence of N1 pathological lymph nodes (Hilar 10/11, Lobar 12, Sublobar 13/14)
- Incidence of N1 pathological lymph nodes (Hilar 10/11, Lobar 12, Sublobar 13/14) in patients with pathological evidence of visceral pleural invasion

Secondary Outcomes

Overall survival (1-3-5 years)

Disease free-survival (1-3-5 years)

Tumor recurrence (pattern: local, regional, distance)

DFS is defined as the time of surgical intervention to tumor recurrence or death and OS is defined as the time of surgical intervention to death.

The minimum sample size to estimate the incidence was calculated using the method described by Humphrey et al:

$$Sample\ size = \left(\frac{1.96}{I_e * L}\right)^2 * I_e * (1 - I_e)$$

Setting the expected incidence (of patients with an N1 lymph node metastasis to 0.2, and the allowable error of the incidence (L) to 0.2 the minimum required sample size is 385 patients. After doubling the number to account for loss to follow-up and a lower true incidence, the final sample size is 770 patients.

The minimum sample size for the survival analysis was calculated using the method described by Schoenfeld:

$$Events\ needed = \frac{(Z_\alpha + Z_\beta)^2}{(\log(RH)^2 * (1 - q_1) * q_1)}$$

We set the type I error rate to 0.05 (), and the type II error rate to 0.2 (). Based on previous studies the expected percentage of patients with N1 lymph node metastasis was set to 20% (q1) and the relative hazard (RH) was set to 1.5). The number of events needed to achieve a power of 80% in the cohort was 298.
The incidence of N1 lymph node metastasis overall and the incidence of metastasis to the different lymph node stations (Hilar 10/11, Lobar 12, Sublobar 13/14) will be calculated by dividing the number of the respective events by the patient years separately. Ninety-five percent confidence intervals will be calculated using the method described by Ulm (ref ULM).
To investigate the association between visceral pleural invasion and the presence of metastatic lymph nodes univariate and multivariate logistic regression models will be fitted to the data.
For the secondary outcomes, Kaplan-Meier curves for the overall cohort and the subgroups will be created, for the disease-free survival and overall survival separately. Univariate Cox proportional-hazards models will be fitted for each of the predictors of interest. Hazard ratios and corresponding 95% confidence intervals will be calculated from the coefficients. Furthermore, we will also create multivariate Cox models adjusting for age, sex, and relevant comorbidities. The assumption of constant hazards will be checked by calculating the scaled Schoenfeld residuals and inspecting the correlation with time both statistically and visually. The

assumption of noninformative censoring will be tested by creating optimistic models in which all censoring will be set to the longest survival time, too pessimistic models, in which all censored time points will be regarded as events. If the results point in the same direction, we will regard the censoring as noninformative.
All analysis will be performed using the statistical software "R" (Version 4.1.2).

9. Reporting obligations
The ethics committee must be notified of any change of project leader in advance. The completion or discontinuation of the research project must be reported to the ethics committee within 90 days.

10. Data protection
Uncoded data, coding and storage of the key

Only members of the study team will have access to the information. The purpose of sharing data is to increase the ability of the investigators to analyze and translate data into a meaningful report validating their individual findings. Data will be de-identified, coded securely, and stored on a password-protected, virusprotected, firewalled and encrypted Redcap server at Cantonal Hospital Lucerne. The data from the others centers will be sent to us electronically via Email with password protected *.Zip files. There will be no expenses related to the data sharing. Since this protocol is non interventional trial, the only risk to the patient would involve a breach of privacy. This risk is minimized, however, since only indirect identifiers will be recorded at all points throughout the course of the study, ensuring that patient confidentiality is safely kept and the patients will only be recorded in the database by their participant identification code. The link between this code and the patient identity will be in hard copy only and will be stored by the project leader in a locked location separate from the data collections forms. This code document will be destroyed upon completion of the study data extraction. The members of the study team will maintain professionalism at all times, ensuring the full protection of each patient's privacy.

11. Information on the storage of data and samples

All collected data from health records and physician files will be stored in Lucerne for 1 year After completion of the study analysis.

12. Retention period

13. Ethical and regulatory requirements

Risk-benefit assessment:
During an anatomical lung cancer resection along with a segmentectomy/lobectomy or bilobectomy, the gold standard is to perform a radical mediastinal lymphadenectomy.
The mediastinal lymph nodes are routinely screened for metastases but not the intraparenchymal lymph nodes (despite they are always already inside the resected lung specimen).
Discover a link between visceral pleural invasion and metastases in intraparenchymal lymph nodes could dramatically change the management of these patients who actually are not receiving adjuvant chemotherapy because the intraparenchymal lymph nodes are not routinely examined by pathologists.

No risks for the patients could derive from the pathological examination of these lymph nodes

This project complies with the regulatory requirements of the HRA and the HRO. The prerequisite for carrying out the research project is the approval of the competent ethics committee.

14. Results / transparency / publication

If the results of the study will allow meaningful scientific conclusions, they will be presented in scientific meetings and/or used to write scientific publications

15. Funding / Data sharing / Declaration of interest

This research received no specific grant from any funding agency in the public, commercial or not-for-profit sector.
After identifying international centers who would like to be involved in this trial a specific amendment to the ethic committee will be submitted along with a data sharing agreement with each center.
No conflict of interest to declare.

16. References

-Huang H, Wang T, Hu B, Pan C. Visceral pleural invasion remains a size-independent prognostic factor in stage I non-small cell lung cancer. Ann Thorac Surg. 2015 Apr;99(4):1130-9. doi: 10.1016/j.athoracsur.2014.11.052. Epub 2015 Feb 20. PMID: 25704861.

- Bi L, Zhang H, Ge M, Lv Z, Deng Y, Rong T, Liu C. Intrapulmonary lymph node (stations 13 and 14) metastasis in peripheral non-small cell lung cancer. Medicine (Baltimore). 2021 Jul 9;100(27):e26528. doi: 10.1097/MD.0000000000026528. PMID: 34232188; PMCID: PMC8270592.
- Seok Y, Lee E. Visceral Pleural Invasion Is a Significant Prognostic Factor in Patients with Partly Solid Lung Adenocarcinoma Sized 30 mm or Smaller. Thorac Cardiovasc Surg. 2018 Mar;66(2):150-155. doi: 10.1055/s-0036-1586757. Epub 2016 Aug 12. PMID: 27517168.
- Park S, Cho S, Yum SW, Kim K, Jheon S. Comprehensive analysis of metastatic N1 lymph nodes in completely resected non-small-cell lung cancer. Interact Cardiovasc Thorac Surg. 2015
Nov;21(5):624-9. doi: 10.1093/icvts/ivv209. Epub 2015 Aug 4. PMID: 26242319.
- Wightman SC, Lee JY, Ding L, Atay SM, Shemanski KA, McFadden PM, David EA, Kim AW. Adjuvant chemotherapy for visceral pleural invasion in 3-4-cm non-small-cell lung cancer improves survival. Eur J Cardiothorac Surg. 2022 Jun 15;62(1):ezab498. doi: 10.1093/ejcts/ ezab498. PMID: 35325098.
- Fibla JJ, Cassivi SD, Brunelli A, Decker PA, Allen MS, Darling GE, Landreneau RJ, Putnam JB. Re-evaluation of the prognostic value of visceral pleura invasion in Stage IB non-small cell lung cancer using the prospective multicenter ACOSOG Z0030 trial data set. Lung Cancer. 2012 Dec;78(3):259-62. doi: 10.1016/j.lungcan.2012.09.010. Epub 2012 Oct 3. PMID: 23040416;
PMCID: PMC5709090.
- Nitadori JI, Colovos C, Kadota K, Sima CS, Sarkaria IS, Rizk NP, Rusch VW, Travis WD, Adusumilli PS. Visceral pleural invasion does not affect recurrence or overall survival

among patients with lung adenocarcinoma ≤ 2 cm: a proposal to reclassify T1 lung adenocarcinoma. Chest. 2013 Nov;144(5):1622-1631. doi: 10.1378/chest.13-0394. PMID: 23807749; PMCID:
PMC3817930.
